# Artificial Intelligence in Obstetrics and Gynecology: Transforming Care and Outcomes

**DOI:** 10.7759/cureus.64725

**Published:** 2024-07-17

**Authors:** Dharmesh J Patel, Kamlesh Chaudhari, Neema Acharya, Deepti Shrivastava, Shaikh Muneeba

**Affiliations:** 1 Department of Obstetrics and Gynecology, Jawaharlal Nehru Medical College, Datta Meghe Institute of Higher Education & Research, Wardha, IND

**Keywords:** clinical applications, maternal health, gynecology, obstetrics, artificial intelligence

## Abstract

The integration of artificial intelligence (AI) in obstetrics and gynecology (OB/GYN) is revolutionizing the landscape of women’s healthcare. This review article explores the transformative impact of AI technologies on the diagnosis, treatment, and management of obstetric and gynecological conditions. We examine key advancements in AI-driven imaging techniques, predictive analytics, and personalized medicine, highlighting their roles in enhancing prenatal care, improving maternal and fetal outcomes, and optimizing gynecological interventions. The article also addresses the challenges and ethical considerations associated with the implementation of AI in clinical practice. This paper highlights the potential of AI to greatly improve the standard of care in OB/GYN, ultimately leading to better health outcomes for women, by offering a thorough overview of present AI uses and future prospects.

## Introduction and background

Artificial intelligence (AI) has advanced significantly, particularly in the medical field. AI has swiftly become a part of daily life. With the rise of big data collection, the potential applications of AI have expanded, along with expectations. The medical field, in particular, has seen a significant increase in interest regarding AI applications, as shown by the substantial number of research papers published over the past 20 years [1,2]. AI has started to transform the way doctors diagnose, treat, and manage a variety of problems in obstetrics and gynecology (OB/GYN). The use of AI in OB/GYN is examined in this paper, along with its possible advantages, drawbacks, and prospects in this quickly developing discipline. However, there is a big gap between the research papers published and the real use of AI in clinical settings [3,4].

In the 1960s, AI was first introduced to simulate human intelligence in complex devices like computers. Technological advancements in machine learning (ML) and deep learning have propelled the progress of AI [5]. ML involves algorithms designed to process data and enables machines to learn independently without explicit programming. These models can autonomously adapt and enhance their performance based on the data they process. ML algorithms are categorized into supervised and unsupervised types. Supervised learning includes regression (such as basic regression methods) and classification (such as decision trees and support vector machines). Algorithms for unsupervised learning utilize random samples to discover patterns or similarities among them. The two subcategories of these techniques are dimensionality reduction and clustering (for example, K-means or Gaussian mixture models). Clustering is typically used when the learning target is known in advance, while dimensionality reduction serves as an exploratory strategy where the final goal is determined after analysis [6]. The two subcategories of these techniques, dimensionality reduction and clustering, play crucial roles in data analysis and pattern recognition within the realm of AI. Dimensionality reduction techniques like PCA, t-SNE, and autoencoders aim to distill essential features from data while reducing noise and redundancy. PCA identifies key components explaining data variance; t-SNE visualizes high-dimensional data in lower dimensions; and autoencoders compress data representations.

Clustering methods such as K-means and GMM group similar data points into clusters without predefined labels. K-means assigns points to clusters based on centroid proximity, while GMM models data as Gaussian distributions for probabilistic clustering.

Dimensionality reduction is exploratory and useful for visualizing or preprocessing complex data. Clustering discovers patterns in data where the structure or cluster count is unknown. Both are vital in fields from healthcare to finance for extracting insights from large datasets.

Deep learning, a specialized branch of ML, utilizes artificial neural networks (ANNs) to analyze and interpret large volumes of data. These networks consist of numerous neural nodes arranged similarly to neurons in the human brain [7]. ANNs are robust mathematical systems capable of interpreting complex data. Due to their many connections, neurons can communicate with one another and determine the most plausible responses. This interconnected structure allows computers to mimic cognitive processes like inference, helping to determine the most likely solutions to problems. This advanced form of AI is applied in medicine to analyze large datasets, assisting with disease monitoring, diagnosis, and prevention. Convolutional neural networks (CNNs) are employed in deep learning for tasks related to video and image processing. These networks have deeper topologies with more convolutional layers, enhancing their ability to integrate and process visual data effectively [8].

Until 2020, the US FDA had approved only 20 AI-based medical innovations [9]. However, FDA approvals for AI applications in this field have not yet been granted. Moreover, Web of Science indicates a significant disparity in the number of articles on AI used in OB/GYN between core discipline journals for AI/computer science (82%), and OB/GYN (18%). Generally, OB/GYN journals delay publishing research on AI until its therapeutic utility is well proven [10]. Many journals have integrated AI into clinical decision-making in pregnancy, covering themes such as fetal MRI scans using AI, prediction of preterm labor using electrohysterography, and assessment of fetal distress risk during labor [11-13]. Furthermore, broader research has explored factors within the healthcare sector influencing the real-world adoption of AI [14-19]. However, there is a lack of studies investigating obstetrical clinicians’ perspectives on the potential contributions of AI within their field. This gap is concerning because understanding the expectations and needs of individual healthcare professionals is crucial for successfully integrating e-health technologies into clinical practice [20].

Additionally, there may be a discrepancy between the goals of AI research and the needs of physicians if the majority of OB/GYN research is conducted and published within the AI community. Healthcare professionals need to stay informed about the latest developments in AI applications for obstetrics and should play an active role in guiding the direction of this emerging field of study.

To bridge the gap between research and clinical practice, it is crucial to understand the expectations of obstetrical practitioners and identify promising areas for AI application development within their sector. This understanding will ensure that AI research meets the needs of its users, namely doctors and ultimately patients. Therefore, the aim of this research is to address the following questions: What are obstetric clinicians’ perceptions of AI, and where do they believe AI could be beneficial in patient treatment?

The types of studies included in this article are primarily review articles and research studies focusing on the application of AI in OB/GYN. These studies explore various AI technologies, such as ML algorithms and deep learning models like CNNs, and their impact on diagnostic imaging, predictive analytics, personalized medicine, and clinical decision-making within the field of OB/GYN.

## Review

Importance of AI in OB/GYN

The importance of AI in OB/GYN AI has been utilized in medicine for decades. One of the early instances was MYCIN in the 1970s, developed at Stanford University by Edward Shortliffe’s team. It could diagnose bacterial infections and suggest appropriate antibiotics, showcasing potential in medical decision-making. In the 1980s, systems like DXplain at Massachusetts General Hospital assisted in disease diagnosis. These early AI systems relied on rule-based approaches but had limited capabilities. Another early application was AI-based diagnostic systems for ultrasound (US) images in the 1970s and 1980s, aiding radiologists in spotting fetal anomalies [21].

Recently, AI in OB/GYN has seen renewed interest, leveraging advances in ML and data abundance. AI now excels at analyzing imaging data such as US and MRI scans, automatically identifying structures like the placenta or fetal organs. Predicting preterm birth is another focus, using ML to analyze electronic health records (EHRs) for patterns associated with this outcome, improving prediction, and guiding clinical decisions. AI has also been instrumental in the real-time monitoring of high-risk pregnancies, analyzing fetal heart rate data to detect patterns linked with fetal distress.

Diagnostic imaging and interpretation

AI has shown remarkable capabilities in improving diagnostic imaging, particularly in US and MRI.

US Imaging

One safe, noninvasive technique for diagnosing pregnancies is US. Yet, despite its broad application, it can be challenging to obtain accurate readings in particular situations, including motion distortions, hazy borders, acoustic shadows, low signal-to-noise ratio, maternal obesity, and speckle noise, which make precise readings challenging [22].

ML has been used for a number of years to help with the automatic recognition and distinction of different fetal body parts through algorithms on US images of fetuses. Algorithms for obtaining and measuring biometric data and fetal features from US pictures have been developed in a number of research studies [23]. For the time being, there is a semi-automated application for interpreting fetal ultrasonography; if a sonographer or doctor chooses the right pictures of each body component, the program employs an AI algorithm to automatically generate body measurements. Many businesses are getting ready to offer services relating to this technology, which is already in use. For example, automated standard scan planes have been established for quantifying fetal biparietal diameter and head circumference using three-dimensional transthalamic plane US pictures and two-dimensional transventricular US images of the fetal brain [24,25].

Further studies have demonstrated the efficacy of ML in recognizing embryonic organs and structures, which helps diagnose congenital anomalies [26-31]. A summary of research on deep learning applications in fetal biometry and comprehensive fetal imaging, encompassing features like the heart and cervical length, is shown in Table 1.

**Table 1:** Deep learning research on obstetric ultrasonography [37] AI, artificial intelligence; AUC, area under curve; CNN, convolutional neural network; FL, femur length; FHR, fetal heart rate; GA, gestational age; HC, head circumference; ML, machine learning; TPR, true positive rate

Study	Year	Field	Total number of patients/images	AI technology	Outcomes
Burgos-Artizzu et al. [31]	2020	Fetal anatomical planes: brain, thorax, abdomen, femur, and-cervix	1,792 patients; 12,400 images	CNN	Similar performance to humans, although with restricted detail in plane categorization
Sulas et al. [32]	2021	FHR	25 patients; 43 images; 174,319 pulsed-wave Doppler segments	7 envelope tracing techniques and 23 processing steps	98% accuracy
Arnaout et al. [33]	2021	Fetal heart imaging between 18 and 20 weeks GA	107,823 images	Deep learning segmentation model	Sensitivity: 95%; specificity: 96%; AUC: 0.99
Bahado-Singh et al. [34]	2019	Cervical length (<15 mm) combined omics, demographic, and clinical data	26 patients	Comparison of six ML techniques with deep learning performing best	AUC: Delivery <28 weeks gestation after amniocentesis: 0.890; delivery <34 weeks gestation: 0.890; NICU admission: 0.792
Sciortino et al. [35]	2017	Nuchal translucency	12 patients; 382 frames	Wavelet and multi-resolution analysis	64% having an error of 1 pixel or less TPR 99.5%
Papageorghiou et al. [36]	2016	GA estimation	4,229 patients	Generic algorithm	In the third trimester, accurate estimations of GA by FL and HC

MRI Interpretation

In gynecology, AI aids in interpreting MRI scans for conditions like endometriosis, fibroids, and ovarian tumors. AI-driven image analysis helps differentiate between benign and malignant masses, thereby aiding in early and accurate diagnosis. In obstetrics, MRI is a subject of active research alongside US. MRI is frequently used to distinguish various fetal brain conditions and evaluate the severity of placenta previa. For example, a particular study involved the automated extraction and analysis of fetal brain structures from MRI scans of 45 pregnant women, including automated volume measurements [12]. Another study utilized various AI techniques to analyze 59 MRI scans of fetuses with ventriculomegaly, predicting the need for postnatal interventions like cerebrospinal fluid diversion with 91% accuracy [38]. This demonstrates AI’s potential not only in diagnosing conditions using MRI but also in predicting necessary treatments.

Furthermore, AI applications in MRI have been extensively studied for placental conditions. AI techniques accurately diagnosed placental adhesions in 99 pregnant women diagnosed with placenta previa, achieving 100% sensitivity, 88.8% specificity, and 95% accuracy [39]. In another study, 44 pregnant women (including twins) underwent an MRI scan to map vessel distribution on the placental surface and measure placental volume [40]. These findings are crucial for the diagnosis and management of twin-to-twin transfusion syndrome.

Predictive analytics and risk assessment

AI can predict pregnancy-related complications and assess risks with considerable accuracy.

Preterm Birth Prediction

AI is emerging as a promising tool to tackle this complex issue by enabling early prediction, personalized risk assessment, and improved management strategies. By integrating multiple variables, AI models can predict the likelihood of preterm birth more accurately than traditional methods, aiding in the early identification of high-risk pregnancies. AI-powered analysis of biological markers (e.g., cytokines and cervical length) and imaging techniques (e.g., US and MRI) enhances the assessment of maternal-fetal health. Algorithms interpret subtle changes in biomarkers and imaging data to detect early signs of preterm labor or complications such as cervical insufficiency, providing clinicians with actionable insights for proactive management [41].

AI-driven fetal monitoring systems analyze real-time data from sensors and monitors to assess fetal well-being. These systems detect deviations from normal fetal patterns, such as heart rate variability or uterine contractions, prompting timely interventions to prevent preterm labor or optimize neonatal outcomes. Models analyze various factors, including maternal age, medical history, and biomarkers, to predict the risk of preterm birth. Early identification allows for timely interventions to prevent or manage preterm labor.

Preeclampsia

AI algorithms utilize maternal health data to identify women at high risk of developing preeclampsia, facilitating closer monitoring and early detection. AI-enhanced imaging techniques aid in assessing placental health and blood flow, which are critical in managing preeclampsia and ensuring optimal fetal growth. AI algorithms analyze extensive datasets, incorporating maternal demographics, medical history, biochemical markers (e.g., serum levels of angiogenic factors like sFlt-1 and PlGF), and imaging data (e.g., Doppler US of uterine arteries). These models identify patterns and factors associated with preeclampsia risk, enabling the early identification of high-risk pregnancies [42].

Gestational Diabetes

AI technologies are being explored and implemented to enhance the detection, monitoring, and management of gestational diabetes mellitus (GDM), offering promising avenues for improving outcomes for both mothers and babies. Large patient data sets can be analyzed by ML algorithms to find risk factors linked to the development of GDM. To estimate the chance of GDM starting, these algorithms can incorporate clinical information such as the mother’s age, BMI, prior obstetric history, and glucose levels. By identifying high-risk pregnancies earlier, healthcare providers can intervene promptly with preventive measures and personalized management plans. AI also plays a crucial role in continuous glucose monitoring and management. Advanced AI algorithms can analyze real-time data from continuous glucose monitors worn by pregnant women with GDM. These algorithms can detect patterns and trends in glucose levels, providing timely insights to both patients and healthcare providers.

AI-powered decision support systems can recommend personalized dietary adjustments, insulin dosages, or lifestyle modifications based on individual glucose profiles, optimizing glycemic control throughout pregnancy. Furthermore, AI-driven predictive models are being developed to forecast complications associated with GDM, such as macrosomia (large birth weight), neonatal hypoglycemia, and preterm delivery. By analyzing diverse datasets and incorporating factors such as maternal glucose levels, fetal growth parameters, and maternal health indicators, these models aim to improve risk stratification and guide clinical decision-making to mitigate adverse outcomes. In clinical practice, AI-enabled tools are also being utilized to streamline workflows and enhance efficiency in GDM management. For instance, AI-powered EHR systems can automate data entry, flag abnormal trends in glucose levels for review, and provide decision support alerts to healthcare providers. This integration not only reduces the burden on healthcare professionals but also ensures timely interventions and continuity of care for pregnant women with GDM.

Personalized treatment plans

AI enables the development of personalized treatment plans tailored to individual patients’ needs.

Treatment of Infertility

In the realm of in vitro fertilization (IVF), one promising application of AI involves the identification of the most viable oocytes and embryos. One research has explored using AI systems that combine texture analysis techniques, such as local binary patterns extracted from images, with ANNs. Their findings have demonstrated superior performance compared to conventional methods, offering a noninvasive and objective approach to selecting optimal oocytes and embryos. This technology underscores its potential advantages in embryo selection, particularly in regions where regulations prohibit sex-based embryo selection. Moreover, AI algorithms can analyze data from IVF cycles to identify key factors associated with improved implantation and pregnancy rates, thereby enhancing the customization of IVF protocols for individual patients.

Gynecologic Oncology

In cancer treatment, AI can help tailor chemotherapy and radiation therapy plans based on the genetic profile of the tumor, improving outcomes and minimizing side effects. AI algorithms analyze medical imaging data, such as US, MRI, and CT scans, to detect early signs of gynecological cancers. These systems can identify subtle abnormalities that may indicate the presence of tumors or precancerous lesions, enabling earlier intervention and improved survival rates. AI enhances the analysis of tissue samples (biopsies) through digital pathology and histopathological image analysis. ML algorithms can assist pathologists in identifying cancerous cells with greater accuracy and efficiency, reducing diagnostic errors and providing more timely treatment recommendations [43].

Use of AI in OB/GYN

Fetal Cardiotocography (CTG)

An example of AI’s usefulness is in evaluating cardiotocographs during labor. Inconsistencies in intrapartum monitoring across different centers and among obstetricians are common issues. AI could provide more consistent assessments, lowering the risk of perinatal and maternal complications. Additionally, intelligent support software could decrease the likelihood of legal disputes and alleviate the financial strain on healthcare systems, especially in developing countries. AI has been utilized in CTG analysis in systems like the Computer-Aided Fetal Evaluator and the INFANT study protocol. These advanced systems use complex algorithms to address challenges in CTG analysis [31,44]. Another similar technology is System 8000, which accounts for episodic changes in fetal heart rate and movements typical of sleep states. It records various parameters, such as CTG quality, uterine contraction peaks, baseline heart rate, variability, decelerations, and accelerations [45].

According to a recent meta-analysis, continuous CTG monitoring was linked to a 50% decrease in newborn seizures [46]. Numerous studies, including both retrospective cohort studies and randomized controlled trials, have examined AI in CTG interpretation since the first machine-based CTG interpretation study in 1989. About 50,000 patients were enrolled in three of these randomized controlled studies, and the results on risk identification and lowering unfavorable outcomes were not always consistent.

An overview of ML analyses of CTG for predicting neonatal outcomes is presented in Table 2.

**Table 2:** Overview of ML analyses of CTG for predicting neonatal outcomes 2D, two-dimensional; AI, artificial intelligence; ARM, artificial rupture of membranes; AUC, area under curve; CNN, convolutional neural network; CTG, cardiotocography; GA, gestational age; HIE, hypoxic-ischemic encephalopathy; ML, machine learning

Study	Year	Number of patients	AI technology	Inclusion criteria	Outcomes
Liu et al. [47]	2021	3,239	Fully convolutional network	More than 36 weeks GA; single intrauterine live fetus	It exhibits greater sensitivity in predicting fetal compromise but also shows a higher false positive rate compared to clinical practice.
Ogasawara et al. [48]	2021	324	CNN model	Umbilical artery pH <7.20 or an Apgar score <7 at one minute	AUC: 0.73 ± 0.04; early detection of a compromised fetus
Zhao et al. [49]	2018	552	Eight-layer deep 2D CNN	-	Categorize CTG as normal or pathological
Brocklehurst et al. (the INFANT trial) [50]	2017	46,042	Infant-K2	More than 16 years above; single or twin intrauterine live fetuses; more than 35 weeks GA	It effectively identifies abnormal CTG patterns but does not improve clinical outcomes.
Nunes et al. [51]	2017	7,730	Omniview-SisPorto	More than 18 years above; single intrauterine live fetus; more than 36 weeks GA	Although a low rate of acidosis was observed, there was no statistically significant reduction in obstetric interventions or acidosis.
Ignatov and Lutomski [52]	2016	720	Quantitive CTG decision support system: Nexus-obstetrics	More than 16 years above; single intrauterine live fetus	Reduced risk in the interventional ARM compared to control
Georgieva et al. [53]	2014	22,790	-	Acidosis (pH <7.5) severe complications (stillbirth, HIE, NICU admission, or neonatal death)	Enhanced sensitivity and a lower false positive rate in detecting acidosis or severe compromise compared to conventional clinical practices.
Warrick et al. [54]	2009	220	Support vector machine	HIE; death base deficit of more than 12 mmol	It detected 50% of pathological cases with a 7.5% false positive rate.

Additionally, an analysis of 2,126 CTG exams identified 21 diagnostic parameters. A 2019 study utilizing a deep learning-driven method for automatic CTG classification achieved a sensitivity of 99.716%, a specificity of 97.500%, and an accuracy of 99.503% [55]. Another study, using CTG data from 552 labor cases, developed an algorithm to predict the risk of umbilical cord blood pH ≤7.15, resulting in an accuracy of 98.34%, sensitivity of 98.22%, and specificity of 94.87% [56]. However, a recent systematic review found that ML applications in labor did not improve neonatal outcomes compared to expert interpretation. These outcomes included neonatal acidosis, low cord blood pH, low Apgar scores, mode of delivery, NICU admission, neonatal seizures, and perinatal death [57,58]. This limited effectiveness might be partly due to the fact that ML models for CTG were trained based on human interpretation. Therefore, feature engineering theory has explored an alternative strategy that excludes human interpretation or guidance in system development (Table 2). These advancements suggest that future software capable of automatically analyzing CTG and alerting physicians to potential risks using advanced computer systems may soon be available for clinical application.

Labor Management

AI algorithms analyze maternal health data, including medical history, prenatal tests, and vital signs, to predict labor outcomes and identify potential complications. These predictive models assist healthcare providers in assessing the risk of preterm labor, fetal distress, or other adverse events, allowing for proactive management strategies. AI models predict the timing and progression of labor based on factors such as cervical dilation, uterine contractions, and fetal heart rate patterns [59]. This information helps healthcare providers anticipate the onset of active labor, optimize timing for interventions like labor induction or cesarean section, and reduce unnecessary interventions.

Fetal Monitoring

AI-powered fetal monitoring systems analyze data from multiple sources, including CTG, fetal US, and maternal-fetal physiological parameters. These systems identify patterns and deviations in fetal heart rate, movements, and uterine contractions, offering real-time insights into fetal health. AI algorithms can detect early signs of fetal distress or abnormalities, such as intrauterine growth restriction, abnormal fetal heart rate patterns, and umbilical cord issues [60].

Maternal Health Monitoring

AI assesses maternal health by analyzing vital signs (e.g., blood pressure and heart rate), laboratory tests (e.g., blood glucose levels and proteinuria), and biomarkers (e.g., placental growth factor and cytokines). Real-time monitoring and trend analysis can detect early signs of maternal complications such as preeclampsia, gestational diabetes, or infections, allowing for timely interventions. AI-powered remote monitoring technologies enable continuous surveillance of maternal health parameters outside of clinical settings. Telemedicine platforms with AI support facilitate virtual consultations, remote monitoring of high-risk pregnancies, and prompt interventions, thereby enhancing access to specialized care and improving patient outcomes [61].

Postpartum Care

AI evaluates maternal health by analyzing vital signs (e.g., blood pressure and heart rate), lab results (e.g., blood glucose levels and proteinuria), and biomarkers (e.g., placental growth factor and cytokines). This real-time monitoring and trend analysis can detect early signs of complications such as preeclampsia, gestational diabetes, or infections, allowing for prompt intervention. AI-powered remote monitoring technologies provide continuous surveillance of maternal health parameters outside of clinical environments. Additionally, telemedicine platforms enhanced by AI support virtual consultations, remote monitoring of high-risk pregnancies, and timely interventions, improving access to specialized care and patient outcomes [60].

Challenges 

Data security and privacy are issues that are brought up by the use of AI in healthcare. Maintaining trust and adhering to laws such as the Health Insurance Portability and Accountability Act (HIPAA) requires making sure that patient data is shielded from breaches and misuse [62]. Integrating AI into current clinical practice can be challenging. It requires a significant infrastructure overhaul, education for medical staff, and a departure from established OB/GYN practices. Ethical considerations are also paramount. Issues such as informed consent, transparency, and the potential biases in AI algorithms must be carefully addressed. Upholding patient rights and ensuring equitable treatment are essential to the ethical development and deployment of AI applications in healthcare.

Limitations

AI in medicine faces several challenges, each impacting its effectiveness and ethical considerations.

Insufficient Data

Effective training and testing of AI models requires extensive data, which may be lacking in fields like gynecology, hindering accurate model development.

Data Bias

AI models trained on biased data may produce unreliable predictions, particularly for specific patient groups, limiting their applicability.

Interpretability Issues

Many AI models operate as “black boxes,” making it challenging for healthcare professionals to comprehend their decision-making processes and trust their outputs. Uncertainty handling: AI often struggles with predicting outcomes in uncertain scenarios or when multiple possibilities exist, which is critical in medical diagnoses reliant on pattern recognition.

Ethical Implications

Concerns include potential discrimination and the ethical dilemmas arising from AI potentially replacing human doctors in clinical decision-making.

Future directions

Ongoing research aims to develop more sophisticated AI models with improved accuracy and reliability, leveraging larger datasets and advanced algorithms to enhance diagnostic and therapeutic capabilities. The integration of AI with genomic data in OB/GYN holds promise for personalized medicine, enabling more precise risk assessments, early detection of genetic disorders, and tailored treatment plans based on individual genetic profiles. Future applications of AI in OB/GYN may include remote monitoring and telemedicine, facilitating continuous patient care and management, particularly in underserved or remote areas.

## Conclusions

The review article on AI in OB/GYN underscores substantial advancements and potential benefits in enhancing diagnostic accuracy, predicting pregnancy-related complications, and personalizing treatment plans. It highlights AI's transformative impact on improving maternal and fetal health outcomes through innovative technologies such as advanced imaging analysis, predictive analytics, and remote monitoring systems. This evolution is encapsulated in the phrase “AI in gynecology: where precision meets intuition, algorithms tackle the mysteries of the womb with binary brilliance and a touch of digital dexterity.” Addressing challenges in data privacy, integration into clinical practice, and ethical considerations is essential for maximizing AI’s role in revolutionizing gynecological care worldwide.
